# Tobacco biomarkers and genetic/epigenetic analysis to investigate ethnic/racial differences in lung cancer risk among smokers

**DOI:** 10.1038/s41698-018-0057-y

**Published:** 2018-08-22

**Authors:** Sharon E. Murphy, Sungshim Lani Park, Silvia Balbo, Christopher A. Haiman, Dorothy K. Hatsukami, Yesha Patel, Lisa A. Peterson, Irina Stepanov, Daniel O. Stram, Natalia Tretyakova, Stephen S. Hecht, Loïc Le Marchand

**Affiliations:** 1 Masonic Cancer Center, University of Minnesota, Minneapolis, MN 55455 USA; 2 Norris Comprehensive Cancer Center, University of Southern California, Los Angeles, CA 90089 USA; 3 Cancer Research Center of Hawaii, University of Hawaii, Honolulu, HI 96813 USA

## Abstract

The Multiethnic Cohort Study has demonstrated that African Americans and Native Hawaiians have a higher risk for lung cancer due to cigarette smoking than Whites while Latinos and Japanese Americans have a lower risk. These findings are consistent with other epidemiologic studies in the literature. In this review, we summarize tobacco carcinogen and toxicant biomarker studies and genetic analyses which partially explain these differences. As determined by measurement of total nicotine equivalents in urine, which account for about 85% of the nicotine dose, African Americans take up greater amounts of nicotine than Whites per cigarette while Japanese Americans take up less. There are corresponding differences in the uptake of tobacco smoke carcinogens such as tobacco-specific nitrosamines, polycyclic aromatic hydrocarbons, 1,3-butadiene, and other toxic volatiles. The lower nicotine uptake of Japanese Americans is clearly linked to the preponderance of low activity forms of the primary nicotine metabolizing enzyme CYP2A6 in this ethnic group, leading to more unchanged nicotine in the body and thus lower smoking intensity. But the relatively high risk of Native Hawaiians and the low risk of Latino smokers for lung cancer are not explained by these factors. The possible role of epigenetics in modifying lung cancer risk among smokers is also discussed here. The results of these published studies may lead to a better understanding of susceptibility factors for lung cancer in cigarette smokers thus potentially identifying biomarkers that can detect those individuals at highest risk so that preventive approaches can be initiated at an early stage of the lung cancer development process.

## Introduction

Epidemiological studies demonstrate that the lung cancer risk of cigarette smokers varies by ethnic/racial group, as reviewed here. These differences in risk may be driven by variations in uptake of carcinogens and toxicants in cigarette smoke, as well as genetic and epigenetic factors. In this review, we present mechanistic studies which use biomarkers of nicotine and carcinogen metabolism and uptake to partially explain the observed ethnic/racial differences in lung cancer risk. The biomarkers discussed are well-established in numerous studies in smokers and animal models as excellent measures of carcinogen exposure, and will potentially be useful tools in a precision medicine approach to the identification of smokers at the highest risk of lung cancer. The focus of this review is the use of these biomarkers to better define some of the contributors to the ethnic/racial differences in lung cancer risk. Other factors such as molecular changes in the lung, and underlying differences in somatic mutations, are beyond the scope of the review.

The World Health Organization has defined a biomarker as “any substance, structure or process that can be measured in the body… and influence or predict the outcome of disease”.^1^ The quantitation of the lung cancer risk of smokers depends on an accurate measure of smoking dose and duration. Historically this measure has been pack years, the product of duration of smoking and cigarettes per day (CPD).^2^ However, arguably a better measure of exposure than CPD would be biomarkers of nicotine and/or carcinogen and toxicant uptake, especially if they reflect long-term exposure. These biomarkers include nicotine and carcinogen metabolites, as described here. In support of this approach, prospective studies of smokers in the Shanghai and Singapore cohorts have found that the major nicotine metabolite, cotinine, and total 4-(methylnitrosamino)-1-(3-pyridyl)-1-butanol (NNAL), an established biomarker of the tobacco specific lung carcinogen, 4-(methylnitrosamino)-1-(3-pyridyl)-1-butanone (NNK), are significantly associated with lung cancer risk after adjustment for self-reported smoking history.^3–5^

The studies reviewed here in the cigarette smokers of the Multiethnic Cohort Study (MEC), a large contemporary prospective epidemiologic study, use biomarkers of exposure and metabolism of nicotine, NNK, polycyclic aromatic hydrocarbons (PAH) and other volatile carcinogens to investigate the mechanism underlying the ethnic/racial differences in lung cancer risk.^6–12^

One strong finding that has emerged is the important role of CYP2A6-mediated nicotine metabolism in predicting smoking intensity (the nicotine uptake from each cigarette), carcinogen exposure and lung cancer risk. This relationship is clearly observed in Japanese Americans due to the high prevalence of low or no-activity *CYP2A6* alleles.^7^ However, decreased CYP2A6 activity may influence smoking and carcinogen exposure in any smoker, and the relationship of nicotine metabolism and *CYP2A6* genotype to the lung cancer risk of smokers of European descent was recently established in the Transdisciplinary Research in Cancer of the Lung consortium genome-wide association study data set.^13^

In summary, the research reviewed here provides a mechanistic explanation for some of the observed differences in lung cancer risk among cigarette smokers from different ethnic groups, thus building a foundation for the potential application of biomarkers and genetic analysis in a precision medicine approach to predict lung cancer susceptibility. The biomarkers used in the studies discussed here are metabolites of nicotine and tobacco carcinogens. However, the potential usefulness of DNA methylation of CpG sites as biomarkers of prior tobacco exposure and lung cancer risk in non-current smokers is also reviewed.

## Ethnic Differences in lung cancer risk due to cigarette smoking

Comparisons of cancer risks among ethnic and racial groups have often been used by epidemiologists to generate clues about risk factors and disease susceptibilities. This approach has particularly been productive in the case of smoking and lung cancer. Ethnic differences in lung cancer risk patterns in relation to smoking were first noted in descriptive studies and in the results of analytical studies conducted in Hawaii, Asia and the West. It was noticed in Hawaii that the mid-1900s rise in lung cancer mortality after the introduction of manufactured cigarettes in the preceding decade was associated with a steeper slope in Native Hawaiians compared to any other ethnic groups.^14^ An ecological study among a random sample of 8636 Hawaii residents further supported a greater lung cancer risk in Native Hawaiians due to smoking. Their lifetime use of cigarettes was similar to that of Japanese smokers, despite their 2-fold greater lung cancer incidence rate.^15^ To formally test these differences in the lung cancer risk associated with smoking in different ethnic groups in Hawaii, a population-based case-control study was conducted with 740 cases and 1616 controls. After adjusting for lifetime smoking, education and occupation, Native Hawaiian, Filipino and White male smokers were at 121%, 53% and 46% greater risk of lung cancer, respectively, compared to their Japanese counterparts.^16^ Chinese and Japanese smokers appeared to have a similar risk. These risk patterns were consistent between sexes and across histological types, and were not explained by the type of cigarettes smoked, levels of inhalation or cholesterol and beta-carotene intake. The lower lung cancer risk of Hawaii Japanese and Chinese smokers, compared to Whites, was consistent with the long-noted, 4-10 fold smaller effect size for the smoking and lung cancer association reported for studies conducted in Japan, China and Korea, compared to those conducted in the West.^17–19^

Some evidence for greater lung cancer risk due to cigarette smoking for African Americans compared to Whites was also provided by a small number of case-control studies conducted in the U.S.^20–22^ A case-control study in New Mexico did not show any significant difference in lung cancer risk between Hispanics and Whites after accounting for differences in smoking.^23^

**Fig. 1:**
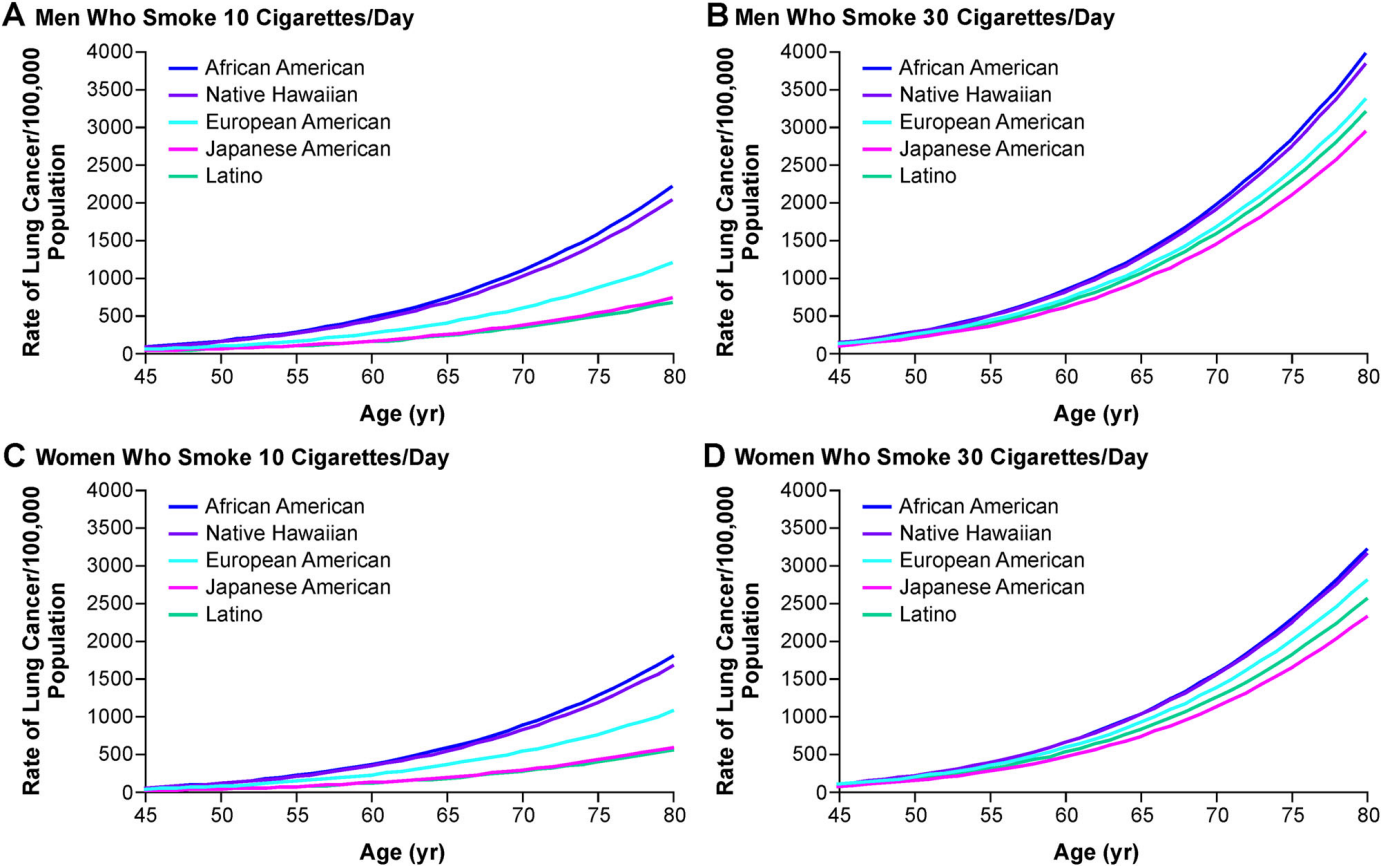
Predicted rates of lung cancer among men who currently smoke 10 CPD **a** or 30 CPD **b** and among women who currently smoke 10 CPD **c** or 30 CPD **d**.^24^ Copyright © 2006 Massachusetts Medical Society. Reprinted with permission from Massachusetts Medical Society

Evidence for the existence of ethnic differences in lung cancer risk associated with cigarette smoking was greatly strengthened and extended by an analysis of the MEC Study.^24^ Lung cancer risk differences were investigated prospectively among nearly 184,000 Japanese-American, African American, White, Latino, and Native Hawaiian male and female residents of Hawaii and Los Angeles. A total of 1979 incident lung cancer cases were identified during the eight-year follow-up. The lung cancer risk differences observed among the five ethnic/racial groups vary based on the number of CPD. Among participants who were light (≤10 CPD) or moderate smokers (11–20 CPD), the risks of African American and Native Hawaiian smokers were significantly greater than those of smokers in the other ethnic/racial groups. Among light smokers and moderate smokers, the relative risks of lung cancer ranged from 0.21 to 0.39 (*P* < 0.001) among Japanese Americans and Latinos, and from 0.45 to 0.57 (*P* < 0.001) among Whites, compared to African Americans.^24^ However, among heavy smokers (>30 CPD), these risk differences were reduced and did not reach statistical significance (Fig. 1). Similar risk patterns were observed for men and women and for each main histologic cell-type of lung cancer.^24^

All these studies assessed cigarette smoking through questionnaires that focused on the age at which the participant started smoking, CPD, duration of smoking, and the number of years since the participant quit smoking to estimate the lifetime exposure of a participant. As a result, ethnic differences between the risk estimates could be due to group differences in self-reporting, or to subtle differences in smoking behavior (e.g., cigarette type, puff size, and depth of inhalation) that are difficult to capture with questionnaires. Errors in self-reported smoking history have been shown to be similar in African Americans and Whites.^25,26^ Menthol cigarettes, which are generally preferred by African American smokers, have not been found to be associated with a greater risk of lung cancer.^22,27,28^

Studies using tobacco carcinogen and toxicant biomarkers have been particularly helpful in better characterizing smoking behavior across ethnic groups. Cotinine, the main metabolite of nicotine, has often been used as a measure of smoking exposure. Data from a large national survey—the Third National Health and Nutrition Examination Survey (NHANES)—have shown that African Americans have higher levels of serum cotinine per cigarette smoked than Whites or Hispanics.^29^ A recent study assessed total nicotine equivalents (TNE)—the sum of nicotine and 6 of its metabolites—as a biomarker of nicotine uptake among smokers in the MEC. After adjusting for age, sex, CPD, creatinine, and BMI, African Americans had significantly higher urinary amounts of TNE than Whites, and Whites had significantly higher TNE levels than Japanese Americans, as described in more detail in the next section.^6^ TNE levels were lower in Native Hawaiians than in Whites and similar in Latinos and Whites. These results from the MEC and NHANES studies showed that African Americans take in more nicotine from each cigarette, and Japanese-Americans take in less nicotine than Whites. Because nicotine uptake can serve as a marker of exposure to tobacco smoke carcinogens (as addressed in the section on carcinogen exposure), these data are consistent with the higher risk for lung cancer in African American smokers and the lower risk for lung cancer of Japanese-American smokers.

In summary, although the data are still relatively limited, especially regarding differences by histological cell types, consistent patterns of ethnic/racial differences in smoking intensity and in lung cancer risk have been described among cigarette smokers. Differences in smoking intensity per reported CPD as measured by TNE relate well to the ordering by cancer risk of African Americans, Whites, and Japanese Americans; however, the lower risk in Latinos and the higher risk in Native Hawaiians evidently cannot be explained using TNE as a measure of intensity. It seems possible that other as yet undetermined pathways to susceptibility are at the root of the high lung cancer risk in Native Hawaiians and relatively lower risk in Latinos. These data should not be interpreted as suggesting that cigarette smoking is less harmful in some groups; however, they offer an opportunity for expanding our understanding of smoking behavior and lung cancer risk and may lead to new approaches to address ethnic/racial disparities in smoking and the risk of lung cancer.

## Nicotine metabolism, CYP2A6 variants, and lung cancer susceptibility

**Fig. 2:**
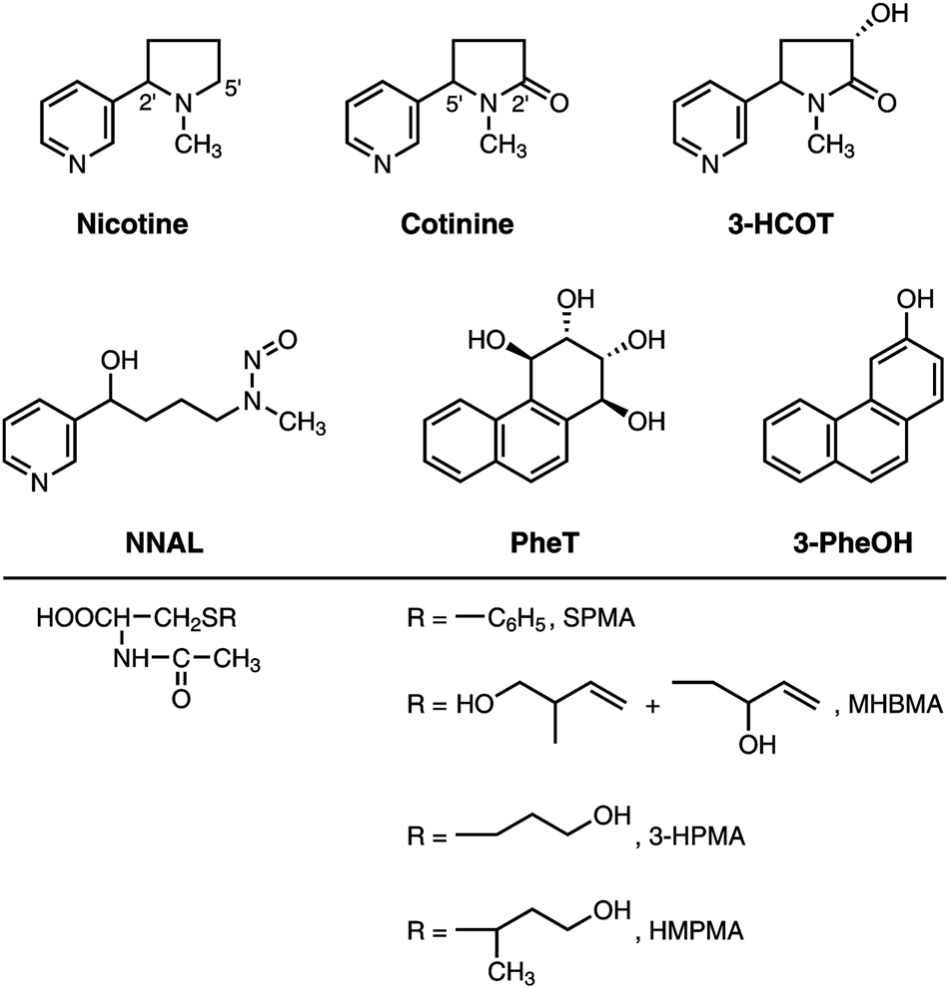
Structures of compounds mentioned in the text

**Fig. 3:**
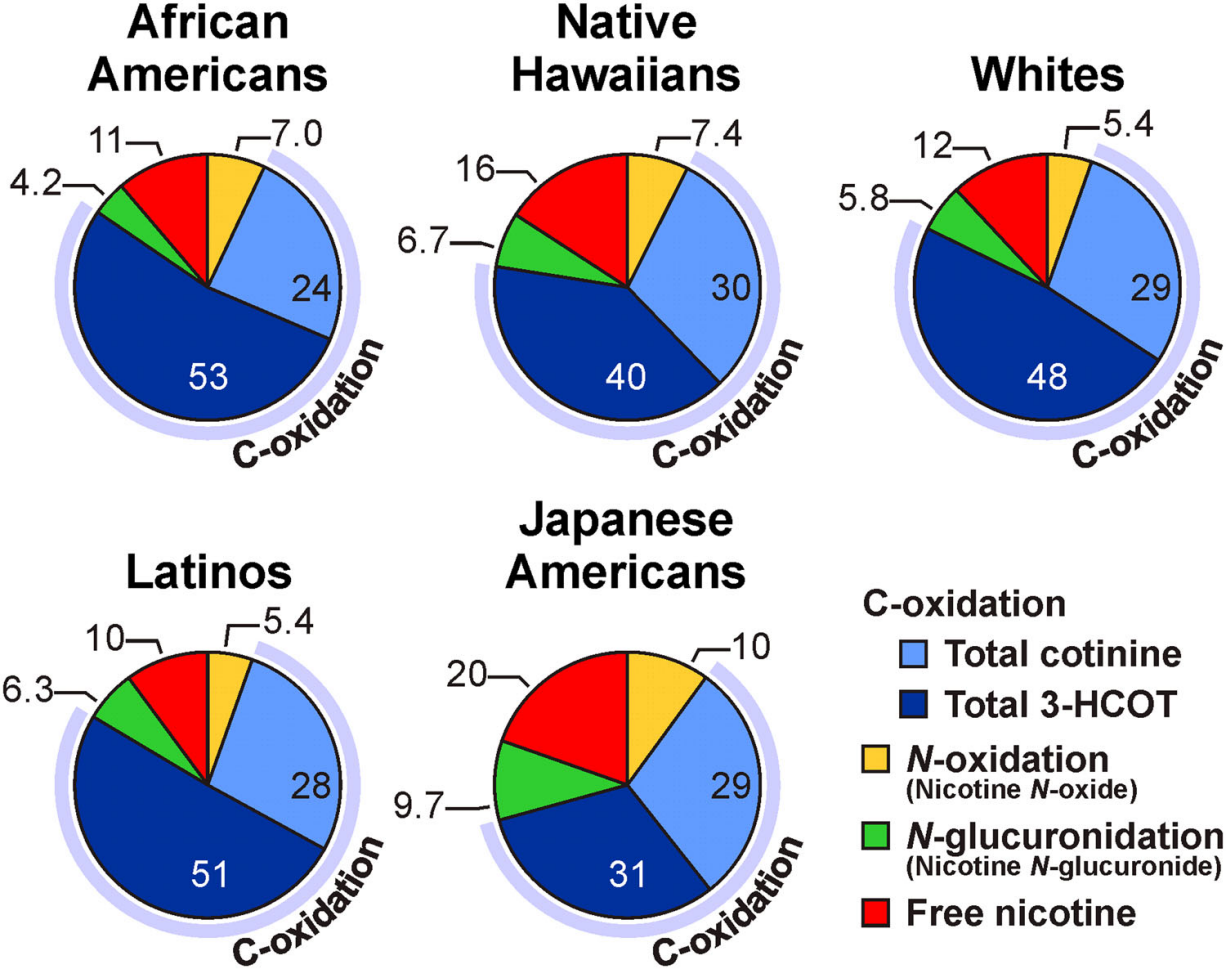
Proportion of nicotine metabolized by C-oxidation, *N*-glucuronidation and *N-*oxidation in five ethnic/racial groups (*n* = 2239 from a subset of the MEC). The values are the molar percent of nicotine and six metabolites excreted in urine, and each slice of the pie is the mean percentage of the compound relative to TNE.^6^ Used by permission of Oxford University Press

Nicotine, the primary addictive agent in tobacco smoke,^30^ is metabolized by three pathways; FMO3-catalyzed *N*-oxidation, UGT2B10-catalyzed *N*-glucuronidation, and CYP2A6-catalyzed C-oxidation.^6,31^ The nicotine C-oxidation pathway is by far the predominant one in most smokers, on average accounting for ~75% of nicotine metabolism. Nicotine C-oxidation to nicotine Δ5’(1’)iminium ion is followed by a second oxidation that generates cotinine, which is further metabolized to *trans* 3’-hydroxycotinine (3-HCOT), a reaction that is catalyzed almost exclusively by CYP2A6 (see Fig. 2 for structures). The sum of urinary nicotine, cotinine, 3-HCOT, and their glucuronides plus nicotine *N*-oxide is referred to as TNE, and accounts for ~85% of the nicotine dose. The unique pattern of nicotine metabolism by the five ethnic/racial groups of the MEC is illustrated in Fig. 3. Japanese Americans, the majority of whom carry one or more *CYP2A6* variant alleles, excrete the greatest amount of unmetabolized nicotine and the lowest proportion of TNE as products of C-oxidation.^6^ The prevalence of non-functional and deletion *CYP2A6* alleles in Asian populations, among whom the reported lung cancer risk for smokers is much lower than it is for Whites, has led to the hypothesis that lower levels of CYP2A6-catalyzed nicotine metabolism (resulting in the presence of more nicotine) causes decreased smoking dose and intensity and therefore decreased carcinogen exposure and lung cancer risk.^32,33^

In both Japanese and White populations, several studies have shown that self-reported CPD are lower for smokers who carry *CYP2A6* variant alleles that code for little or no active CYP2A6 enzyme compared to smokers who carry none of these variant alleles.^34–39^ A genome-wide association study (GWAS) in Japanese reported an association between the haplotype representing the *CYP2A6* deletion (CYP2A6*4) and lower CPD.^38^ Similarly, a GWAS of over 60,000 smokers of European descent found an association between CPD and a SNP in *CYP2A6* that is linked to a variant that codes for a non-functional enzyme, CYP2A6*2.^39^ In addition, *CYP2A6* genotype has been shown to influence smoking intensity (mean and total puff volume).^33^ Therefore, not only may CPD vary with *CYP2A6* genotype, but a smoker’s carcinogen exposure per cigarette may also be influenced.

**Fig. 4:**
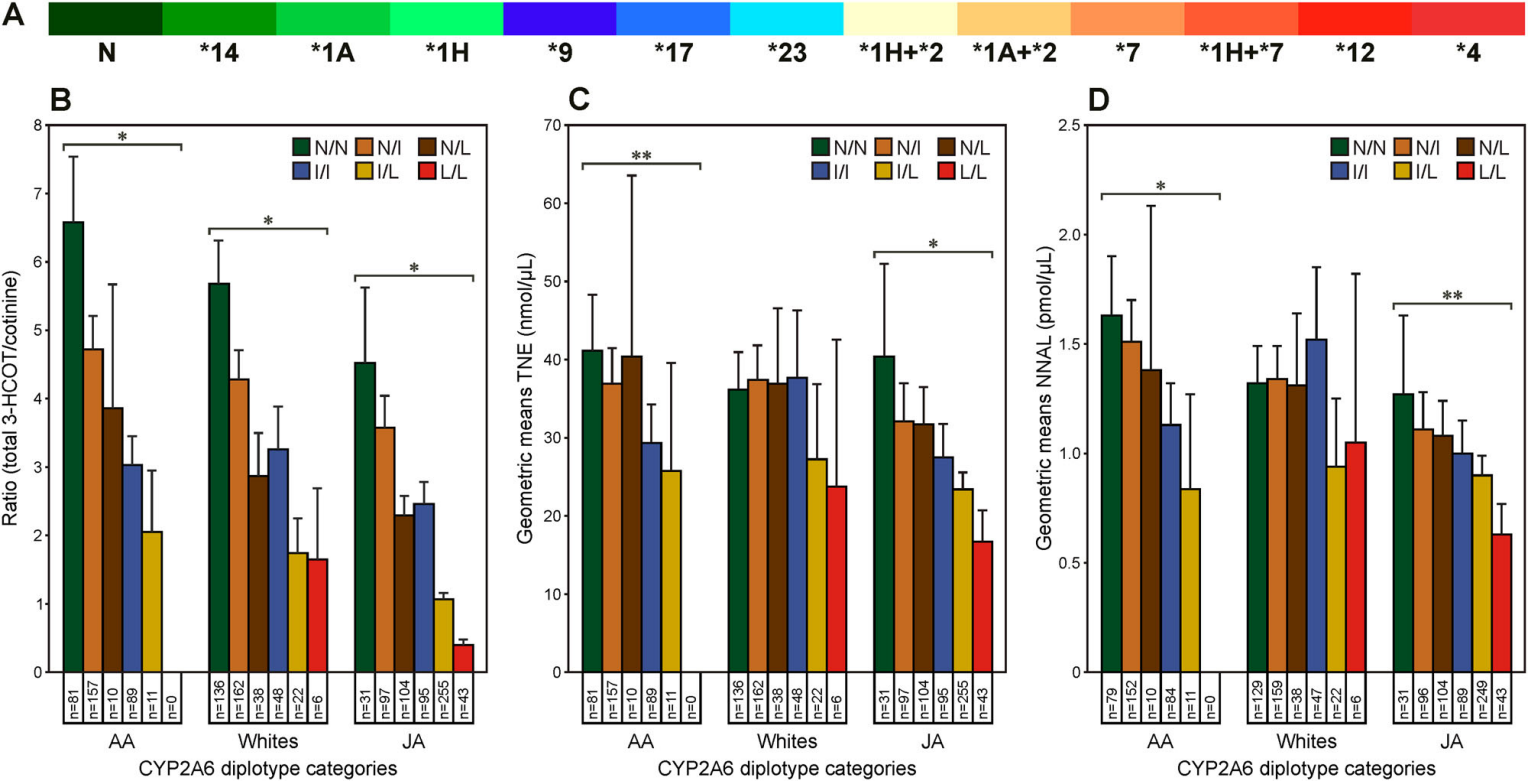
*CYP2A6* halpotypes **a** and diplotypes (**b**–**d**) determined in smokers and their relationship to **b** 3-HCOT/cotinine, **c** TNE, and **d** total NNAL in urine. Haplotypes are listed from left to right in the order of predicted nicotine C-oxidation activity (normal – N, green to none – *4, red) based on reported CYP2A6 activity. Diplotype categories are defined by the functional activity of each allele as follows: N (no variant allele or *1A + *14); I (intermediate activity), *1A, *1H, *9, *17, *23; L (little or no activity), *1H + *2*, *1A + *2*, *7, *1H + *7, *12, *4. The alleles are as described. *P < 0.0001; **P < 0.001 http://www.cypalleles.ki.se/cyp2a6.htm.^7^ Used by permission of Oxford University Press

More than 50 variants of CYP2A6 have been identified, many of which result in decreased nicotine metabolism (see http://www.cypalleles.ki.se/cyp2a6.htm). The relationship of *CYP2A6* genotype to nicotine metabolism and smoking intensity (measured by TNE) has been demonstrated in two lung cancer studies.^7,13,40^ The first was carried out in a Shanghai cohort and examined four variant alleles (CYP2A6*1A, *4, *7, *9, see Fig. 4a) that have a relatively high frequency in Chinese. In this study, there were significantly higher (*P* = 0.002) nicotine levels in smokers predicted by their genotype to have low *CYP2A6* metabolism compared to normal metabolizers.^40^ The second study was carried out in smokers of the MEC; seven additional SNPs were included based on their prevalence in Japanese Americans, African Americans and Whites, and 13 haplotypes were defined (Fig. 4a).^7^ The 68 possible diplotypes defined by the functional activity of each allele were assigned to six categories described in the figure legend (Fig. 4b–d). In all ethnic groups, CYP2A6 activity decreased as expected for these diplotype categories (*P* < 0.0001).^7^ In Japanese Americans, and to a lesser extent in African Americans, the relationship of *CYP2A6* diplotype to nicotine metabolism (as indicated by the total 3-HCOT/cotinine ratio, Fig. 4b) paralleled the relationship to TNE (Fig. 4c) and to the sum of tobacco-specific lung carcinogen metabolite NNAL and its glucuronides (total NNAL) (Fig. 4d). A significant association (*P* for trend) between TNE and *CYP2A6* diplotype for African Americans (*P* = 0.015) and Japanese Americans (*P* < 0.001) was observed. Similarly, urinary total NNAL decreased with CYP2A6 activity for both African Americans (*P* = 0.0008) and Japanese Americans (*P* = 0.0158, Fig. 4d).^7^ Interestingly, TNE values for the Japanese Americans that carry the diplotype with no variant alleles were not different from Whites and African Americans with this diplotype, suggesting the lung cancer risk of these smokers may all be similar. However, in Whites, due in part to the lower frequency of the *CYP2A6* variant alleles determined, there was no significant association of *CYP2A6* diplotype with TNE or total NNAL.

The lung cancer risk of Japanese Americans who reported smoking 30 or fewer CPD in the MEC study was significantly lower than that of Whites while the risk for African Americans was significantly higher. For the current smokers in the MEC, the median CPD for African Americans was 10, for Whites it was 20, and for Japanese Americans 12 (Table 1). In contrast, the median TNE for African Americans, Whites, and Japanese Americans were 44, 36.3, and 27.3 nmol/ml, and urinary total NNAL concentrations were 1.8, 1.19, and 0.914 pmol/ml, respectively^6,8^ (Table 1). These data on TNE and total NNAL are consistent with the relative risk of lung cancer reported for these three ethnic/racial groups. In Japanese Americans, based on the data described above for *CYP2A6* diplotypes (Fig. 4), the lower TNE and total NNAL levels relative to reported CPD appear to be due to the prevalence of low and no-activity *CYP2A6* alleles in this population. Fewer than 10% of the Japanese Americans carried no variant alleles. *CYP2A6* genotype also significantly affected TNE and total NNAL levels in African Americans; however, in this group, 23% carry no variant and none are homozygous for loss of function alleles (Fig. 4).

**Table 1:** CPD and urinary TNE and total NNAL by ethnic/racial group

	Median (25–75%)				
	African Americans (*n* = 364)	Native Hawaiians (*n* = 311)	Whites (*n* = 437)	Latinos (*n* = 453)	Japanese Americans (*n* = 674)
CPD	10.0 (5–15)*	15.0 (9–20)*	20.0 (10–20)	7.07 (4.0–12)*	12.0 (9–20)*
TNE (nmol/ml)^a^	44.4 (27.1–74.0)*	30.3 (19.4–46.8)*	36.3 (21.90–61.5)	32.2 (20.8–53.6)	27.3 (15.8–43.4)*
Total NNAL (pmol/ml)	1.80 (1.01–2.88)*	1.08 (0.66–1.68)*	1.19 (0.72–2.17)*	1.22 (0.70–2.12)*	0.914 (0.532–1.46)*

Adapted from Murphy et al.^6^ (used by permission of Oxford University Press) and Park et al.^8^ (used by permission of American Association for Cancer Research)

*p value for comparison with Whites (*p* < 0.05)

^a^TNE is the sum of urinary total nicotine, total cotinine, total 3-HCOT, and nicotine *N*-oxide

An association of *CYP2A6* genotype and lung cancer has been observed consistently in smokers of Japanese or Chinese descent.^34,41,42^ In some studies the association between lung cancer, smoking and the *CYP2A6* deletion variant, alone or in combination with other reduced function variants, has been linked to CPD.^34,43^ For example, a GWAS that found a significant association between the *CYP2A6* deletion and CPD reported a modest association with lung cancer.^43^ In addition, a nested case control study in Shanghai Chinese reported that, even after adjustment for CPD, *CYP2A6* variants (*4,*7,*9,*1A) were associated with a reduced risk of lung cancer.^40^ However, consistent with the link between nicotine metabolism, smoking intensity and lung cancer, the association was no longer significant after adjustment for TNE.^40^

A lack of statistical power in ethnic/racial groups with lower frequencies of *CYP2A6* null alleles has made establishing an association between *CYP2A6* genotype, smoking dose or intensity, and lung cancer in these groups challenging. This challenge was recently overcome in a large collaborative study that used nicotine metabolism and TNE data from the MEC, and GWAS data from the Transdisciplinary Research on Cancer of the Lung consortium (TRICL).^13^ The TRICL GWAS included 13,479 cases and 43,218 controls of European descent. In the TRICL study, 226 SNPs of the 248 that were associated with CYP2A6 activity in the current smokers of the MEC (at a global significance level of *P* < 5 × 10^-8^) were available. Six of these were associated with a reduced risk of lung cancer at the genome-wide significance level. These data support for the first time in a non-Asian population, the hypothesis that lower CYP2A6 activity leads to less intense smoking and decreased exposure to carcinogens, resulting in a decreased risk of lung cancer.

## Carcinogen exposure and lung cancer susceptibility

Carcinogens form the link between cigarette smoking and lung cancer.^44^ While nicotine is not a carcinogen, each cigarette delivers—along with its dose of nicotine—a complex mixture of at least 70 known carcinogens, some of which cause lung cancer in laboratory animals and are considered to be carcinogenic to humans.^44–46^ Important among these are NNK, PAH such as benzo[*a*]pyrene (BaP), and volatiles such as 1,3-butadiene. All of these compounds readily induce tumors of the lung in mice and/or rats.^47–49^ Other cigarette smoke compounds such as benzene and acrolein, while not known to be lung carcinogens based on studies in laboratory animals, may be excellent monitors for volatile and/or inflammatory agents in smoke.^50,51^ Urinary metabolites can serve as biomarkers to assess individual uptake of these and related carcinogens and toxicants in cigarette smoke. In published studies, biomarkers have been investigated with respect to ethnic differences in lung cancer susceptibility. The structures of these urinary metabolite biomarkers are shown in Fig. 2.

One biomarker that has been evaluated is NNAL, a urinary metabolite of the tobacco-specific lung carcinogen NNK.^52^ NNAL is readily formed by the action of carbonyl reductase and related enzymes on NNK in virtually all biological systems, ranging from cell culture to living humans.^53^ NNAL undergoes glucuronidation at its hydroxyl group to form NNAL-*O*-Gluc or on its pyridine nitrogen to form NNAL-*N*-Gluc. Similar to its parent compound NNK, NNAL is a potent lung carcinogen in rats and mice.^53,54^ Analysis of the same MEC urine samples on which TNE were determined demonstrated that free NNAL as well as “total NNAL” (the sum of free NNAL, NNAL-*O-*Gluc, and NNAL-*N*-Gluc) were highly correlated with TNE in the same samples (*r* = 0.70 for total NNAL and 0.60 for free NNAL).^8^ As shown in Table 1, African Americans had the highest, Whites had intermediate, and Japanese Americans had the lowest levels of total NNAL. As in the TNE data, levels of total NNAL did not fit the lung cancer risk pattern observed for Native Hawaiians and Latinos (Table 1). Overall, the results for urinary total NNAL were consistent with those for TNE and partially reflected lung cancer risk in that the African Americans and Whites had relatively high levels and Japanese Americans had the lowest levels.

Two urinary metabolites of phenanthrene—phenanthrene tetraol (PheT) and 3-hydroxyphenanthrene (3-PheOH)—have also been investigated.^9^ PheT is the end product of phenanthrene metabolism by the diol epoxide pathway, shown to be critical in carcinogenesis by PAH, while 3-PheOH is an accepted monitor of PAH uptake. Both PheT and 3-PheOH were significantly correlated with TNE in the same samples (*r* = 0.47 for PheT and 0.41 for 3-PheOH), although these correlations were not as strong as those for total NNAL and free NNAL. Table 2^9^ presents the geometric means of PheT and 3-PheOH in urine. Amounts of PheT and 3-PheOH were greatest in African Americans and lowest in Japanese Americans (Model 1, see Table 2). The intermediate values in Whites were significantly different from both of these groups. Similar results were obtained upon further adjustment for TNE except that 3-PheOH levels in Japanese Americans were not different from those in Whites, while their amounts of PheT were higher than in Whites. As in the NNAL data, Native Hawaiian and Latino data diverged from the relative risks for lung cancer.

**Table 2:** Geometric means of 3-PheOH and PheT^9^

		Model 1^a^		Model 2^b^	
	*N*	Geometric means	(95% CI)	Geometric means	(95% CI)
3-PheOH (pmol/mL)					
African Americans	358	1.06	(0.984–1.143)^d^	0.85	(0.797–0.901)^d^
Native Hawaiians	321	0.60	(0.559–0.654)^d^	0.62	(0.585–0.664)
Whites	432	0.71	(0.667–0.762)	0.67	(0.639–0.712)
Latinos	448	0.78	(0.734–0.837)^d^	0.77	(0.734–0.816)^d^
Japanese Americans	695	0.59	(0.562–0.626)^d^	0.68	(0.646–0.706)
*p*-value^c^			<0.0001		<0.0001
PheT (pmol/mL)					
African Americans	367	1.30	(1.194–1.422)^d^	0.99	(0.923–1.065)^d^
Native Hawaiians	329	0.86	(0.782–0.941)	0.89	(0.825–0.956)
Whites	443	0.93	(0.858–1.003)	0.87	(0.817–0.926)
Latinos	453	1.14	(1.051–1.228)^d^	1.12	(1.048–1.186)^d^
Japanese Americans	704	0.82	(0.771–0.875)^d^	0.96	(0.916–1.014)^d^
*p*-value^c^			<0.0001		<0.0001

^a^Model 1, adjusted for age, sex, and BMI

^b^Model 2, additionally adjusted for TNE

^c^Global *p*-value

^d^Significant when compared to Whites. Additional adjustment for creatinine in Model 1 abolished the difference between African Americans and Whites in both 3-PheOH and PheT

Further published studies assessed exposures to the carcinogens benzene and 1,3-butadiene by measurement of their urinary mercapturic acids, SPMA and MHBMA, respectively (Fig. 2).^10,11^ Levels of SPMA and MHBMA were highest in African Americans, intermediate in Whites, and lowest in Japanese Americans, essentially replicating the results obtained for the other biomarkers. For SPMA, Native Hawaiians and Latinos had intermediate levels of this urinary metabolite. Both SPMA and MHBMA levels are strongly influenced by the glutathione *S*-transferase (*GSTT1*) genotype, with significantly lower levels in individuals with 0 or 1 copy of the gene compared to those with 2 copies.^10,11,55^ The *GSTT1* null genotype is particularly abundant in Japanese Americans, and in an earlier study of Whites, Native Hawaiians and Japanese Americans the difference in MHBMA levels by ethnic/racial group was only observed in smokers who were *GSTT1* null.^55^ However, in a more recent study, when MHBMA levels were stratified by *GSTT1* copy number, both SPMA and MHBMA levels remained significantly higher in African Americans compared to Whites and were significantly lower in Japanese Americans compared to Whites.^10,11^ Thus, for most of the biomarkers discussed thus far, the highest levels were in African Americans, intermediate in Whites, and the lowest levels were in Japanese Americans, with intermediate levels in Native Hawaiians and Latinos.

Exposures to acrolein and crotonaldehyde by measurement of their urinary mercapturic acid metabolites 3-HPMA and HMPMA, respectively (Fig. 2) have also been reported.^12^ As in the analyses of the other urinary carcinogen and toxicant biomarkers, the levels of these mercapturic acids were significantly correlated with TNE in the same samples (*r* = 0.52–0.6). However, the pattern of urinary 3-HPMA and HMPMA concentrations in the 5 ethnic groups was unique (Table 3).^12^ The highest levels were in Whites and Native Hawaiians, with significantly lower levels in African Americans, Japanese Americans, and Latinos. The lowest levels of both mercapturic acids were in Latinos. While there is scant evidence for the carcinogenicity of acrolein and crotonaldehyde, they are both highly irritating and toxic compounds which produce inflammation and a variety of other effects important in carcinogenesis.^51,56–58^ Furthermore, both can be produced by endogenous processes such as lipid peroxidation, suggesting their possible role in cancer induction among the subjects in this study.

**Table 3:** Geometric means (95% CIs) of 3-HPMA and HMPMA, stratified by race/ethnicity^12^

		Model 1		Model 2	
	*N*	Geometric means	(95% CI)	Geometric means	(95% CI)
3-HPMA (pmol/ml)					
African Americans	362	2623	(2403–2864)^d^	2406	(2226–2600)^d^
Native Hawaiians	329	3689	(3373–4035)	3787	(3499–4099)
Whites	438	3985	(3690–4304)	3549	(3314–3801)
Latinos	449	2087	(1933–2253)^d^	2210	(2066–2365)^d^
Japanese Americans	704	3142	(2956–3340)^d^	3369	(3191–3557)
*p*-value^c^			<0.0001		<0.0001
HMPMA (pmol/ml)					
African Americans	361	2024	(1865–2196)^d^	1860	(1733–1997)^d^
Native Hawaiians	329	2689	(2474–2922)	2759	(2567–2965)
Whites	440	2856	(2659–3068)	2541	(2388–2705)
Latinos	452	1624	(1513–1743)^d^	1720	(1618–1829)^d^
Japanese Americans	702	2108	(1992–2232)^d^	2259	(2150–2373)^d^
*p*-value^c^			<0.0001		<0.0001

^a^Model 1, adjusted for age, sex, creatinine

^b^Model 2, model 1 additionally adjusted for TNE

^c^Global *p*-value

^d^significant when compared to Whites

In summary, published studies demonstrate that the urinary metabolite biomarkers of exposure to the carcinogens NNK, PAH, 1,3-butadiene, and benzene were generally highest in African Americans, intermediate in Whites, and lowest in Japanese Americans, consistent with the TNE data and their relative risks for lung cancer. The urinary biomarkers of exposure to acrolein and crotonaldehyde did not follow this pattern, as the highest levels were found in Whites and Native Hawaiians. These results suggest that endogenous generation of acrolein and crotonaldehyde due to lipid peroxidation and related phenomena may be important in Native Hawaiians and could play a role in their relatively high lung cancer susceptibility.

## Epigenetics and lung cancer susceptibility in smokers

DNA methylation of CpG sites is one of the most commonly studied epigenetic modifications. With the advent of DNA methylation microarrays (i.e., Infinium Human Methylation 27 K or 450 K and EPIC BeadChips), the epigenome-wide association study (EWAS) has become a feasible approach to characterize the epigenome in population studies.^59^ Smoking is a well-established modifier of the epigenome such that differentially methylated DNA sites from smoking may serve as a marker of tobacco smoking and smoking-related lung cancer risk, as well as help to identify the genes involved in lung cancer development. This section reviews the EWAS findings in adults for DNA methylation in relation to smoking traits and lung cancer risk.

Due to the convenience of collecting blood as opposed to other tissue samples, DNA methylation of blood leukocytes remains the most commonly studied tissue for epigenetic modification by smoking. There are at least 20 reported EWAS of smoking traits from blood leukocytes of adults^60–79^ with >2600 CpG sites in >1500 genetic regions identified to be differentially methylated by smoking status. In contrast, the EWAS of smoking in other tissues, e.g., buccal mucosa and nasal epithelium, remains limited.^80–82^ The most frequently replicated smoking-related differentially methylated CpG sites (probes) are the hypomethylation of: cg03636183 in coagulation factor II (thrombin) receptor-Like 3 (*F2RL3*) gene; cg05575921, cg14817490, cg21161138 and cg25648203 in aryl-hydrocarbon receptor repressor (*AHRR)* gene; cg05951221, cg21566642, and cg01940273 in 2q37; cg19859270 in G protein coupled receptor (*GPR15*) gene; and cg06126421 in 6p21.33.

The significance of these known smoking-related hypomethylated CpG sites and others remain unclear. However, many of these genetic regions are associated with cell signaling, metabolism of xenobiotics, and cancer development. Cg03636183 in *F2RL3* was the first smoking-related differentially methylated site identified by EWAS.^60^ *F2RL3* codes for thrombin protease activated receptor 4 (PAR-4), which is expressed in various tissues and plays a key role in platelet activation and cell signaling. Cg05951221 in 2q37 is located near several alkaline phosphatase genes: alkaline phosphatase genes placental (*ALPP*), placental-like (*ALPPL2*) and intestinal (*ALPI*), which are responsible for dephosphorylation of proteins. There are seven genes within the 100 kilobases flanking cg06126421 at 6p21.33 that code for proteins involved in cell cycle check-points, including HLA-B associated transcript (*BAT3*). Genetic variants in *BAT3* have been associated with lung cancer risk in a GWAS.^83^ Another study, however, investigating epigenetic patterns in GWAS-identified candidate genes in lung tumors found that DNA methylation patterns in 6p21.33 did not appear to be significantly different in non-small cell lung cancer tissue compared to adjacent normal tissue (*n* = 34 for discovery; 50 for replication).^84^ *GPR15* is a membrane-localizing protein. A study found hypomethylation of this *GPR15* site was associated with higher *GPR15* RNA expression and expression was highest in current smokers.^85^

The *AHRR* genetic region remains arguably the most consistently and strongly differentially methylated gene, with >35 CpG sites within the body of the gene that have been found differentially methylated in smokers, compared to non-smokers.^77^ Differential methylation patterns of cg05575921, the most frequently replicated probe, located in the intronic region of *AHRR*, have also been associated with multiple smoking traits: CPD, serum cotinine, cumulative amount smoked (pack-years), and time since quitting.^60–75,77,79^ *AHRR* is part of the aryl hydrocarbon receptor (*AHR*) signaling cascade; it mediates dioxin toxicity and is involved in regulation of cell growth and in differentiation and modulation of the immune system. *AHRR* serves as a negative feedback regulator of the *AHR*, which is critical in the metabolism of PAH. The mechanisms by which AHRR methylation alters *AHRR* gene function remain unclear. However, when alveolar cell lines are treated with cigarette smoke condensate an increase in the hypomethylation of transcriptional enhancer markers located adjacent to cg05575921 was found,^86^ suggesting that cg05575921 and other sites in *AHRR* may be actively involved in the induction of gene regulatory elements.

DNA methylation levels from smoking are dynamic.^66,76^ The largest study of DNA methylation change due to smoking cessation (*n* = 745 women) found that sites with greater differential methylation in current smokers were more likely to remain differentially methylated even after >35 years of smoking cessation (e.g., cg05575921 in *AHRR* and cg03636183 in *FRL3*).^69^ If differentially methylated sites can provide additional information beyond self-reported smoking history, they may serve as potential long-term biomarkers of tobacco smoking exposure in former smokers.

DNA methylation studies across race/ethnicity remain sparse and most studies of non-European ancestral populations were conducted in African Americans.^64,67,68,72,77^ The sites with stronger effect sizes in European ancestral populations, e.g., *AHRR* and *F2RL*3, were more likely to be replicated in African Americans.^64,67,68,72,77^ A systematic evaluation of the generalizability of differentially methylated sites by smoking traits across race/ethnicity would help to identify the impact of smoking on the epigenome across populations. The Southall And Brent REvisited (SABRE) cohort in the UK, the only multiethnic study that compared the smoking-related DNA methylation pattern of cg05575921 in *AHRR* by race (South Asian and Whites) among 36 current smokers, found that Whites had lower DNA methylation levels than South Asians (*P* = 2.0 × 10^−3^).^68^ Despite the overwhelming number of studies in blood, tissue specificity of DNA methylation should be considered. Variations in DNA methylation have also been detected within blood cell lines from 20 smokers and 14 nonsmokers; for some of the well-known smoking-related sites, hypomethylation in current smokers was greater in granulocytes followed by monocytes and B-cells.^87^ In an EWAS of pack-years in buccal cells, the most significant findings were detected for the well-established differential smoking-related DNA methylation sites (e.g., *AHRR, F2RL3*) (*P*’s < 10^−^^13^). On the other hand, in an EWAS of current versus former smoking status in buccal cells of chronic obstructive pulmonary disease cases and controls, only modest associations were detected with the well-established sites (*P*’s < 0.05) and the most significant findings were identified in sites previously not associated with current smoking status, with the exception of cg02162897 in cytochrome P450 1B1 (*CYP1B1*).^77^ The association for the well-known sites were not detected in one EWAS of smoking status in nasal epithelial cells and in one EWAS of smoking pack-years in lung tumor cells.^88^ It has been suggested that the nasal epithelium may better reflect the changes in the bronchus as similar genes are expressed in the nasal epithelium and bronchus and the alteration of these genes in various airway diseases appears consistent in both tissues.^89,90^ DNA methylation data has also been used to compute biologic age.^91,92^ Current smokers^93^ and ever smoking lung cancer cases^94^ have been found to have older biologic age.

To date, three EWAS of lung cancer risk using blood cells were conducted in populations of European ancestry (largest sample size *n* = 552 cases).^95–97^ All three studies found cg05575921 in *AHRR*, cg03636183 in *F2RL3* and cg06126421 in 6p21.33 to be associated with lung cancer risk, after adjusting for smoking status and pack-years. One study also detected associations with cg21566642 and cg05951221 in 2q37.1, as well as cg23387569 in 12q14.1.^97^ The latter site was previously found to be only modestly associated with smoking status (*P* = 10^-6^).^77^ Two studies found that the inclusion of any one of the three markers (cg05575921 in *AHRR*, cg03636183 in *F2RL3* or cg06126421 in 6p21.33), improved lung cancer risk prediction as measured by the area under the curve (AUC) by 1-2% (AUC~0.79),^96,97^ and one of these studies found that the improvement in AUC remained even after adjusting smoking status and pack-years.^97^ In another study, the inclusion of methylation values from cg05575921 in *AHRR* and cg03636183 in *F2RL3* was responsible for 37% of the effect of smoking on lung cancer risk.^95^ Only one study was stratified by histologic cell-type and found that the improvements in the AUC were highest in adenocarcinoma (AUC = 0.81) as opposed to squamous cell carcinoma (AUC = 0.79).^96^ These findings suggest that the established smoking-related DNA methylation sites may have utility in lung cancer risk prediction as they may provide important information that is not captured by self-reported smoking history.

Further studies are needed to assess heterogeneity of effects by race/ethnicity, generalize the findings across tissue types, and understand the functional role of differentially methylated sites in relation to disease development. While there are extensive data on differential methylation by smoking status, further investigations of the impact of smoking dose, intensity, and duration on the epigenome of smokers are needed.^79^ Lastly, with the development of newer methylation arrays, specifically the MethEPIC Chip, which includes ~400 K additional CpG sites, predominantly in enhancer regions, we expect novel differentially methylated CpG sites to be identified. Such data may provide additional insights into the influence of smoking on the epigenome and identify genetic regions suitable for smoking-related lung cancer prediction.

Also, in newly initiated studies, we will determine if smoking-related DNA methylation sites have utility in lung cancer risk prediction. DNA methylation data may provide additional insights into the influence of smoking on the epigenome and identify genetic regions suitable for smoking-related lung cancer prediction.

## Conclusions

Epidemiologic studies have clearly shown ethnic/racial differences in susceptibility to lung cancer in cigarette smokers. For the same number of cigarettes smoked, especially at low and moderate levels of smoking as measured by CPD, African Americans and Native Hawaiians have the highest risk for lung cancer, Whites have an intermediate risk, while Latinos and Japanese Americans have the lowest risk. The research described here has partially explained these differences based on uptake of TNE and carcinogens among these groups. African Americans are exposed to the highest levels of TNE and carcinogens, Whites to intermediate levels, and Japanese Americans to the lowest amounts per cigarette. The low exposure of Japanese Americans has been clearly linked to low activity forms of CYP2A6, the primary nicotine metabolizing enzyme. However, other factors are involved in these ethnic/racial differences; current investigations focus on epigenetics and the role of inflammation and oxidative damage in modifying lung cancer risk among these ethnic/racial groups.
